# Test-retest reliability of a new self reported comprehensive questionnaire measuring frequencies of different modes of adolescents commuting to school and their parents commuting to work - the ATN questionnaire

**DOI:** 10.1186/1479-5868-6-68

**Published:** 2009-10-12

**Authors:** Elling Bere, Line A Bjørkelund

**Affiliations:** 1 Faculty of Health and Sport, University of Agder, Kristiansand, Norway

## Abstract

**Background:**

Studies assessing active commuting to school usually use simple questionnaires, and often is mode of commuting reported with a single questionnaire item only. The purpose of the present study is to report the test-retest reliability of a newly developed comprehensive questionnaire on active commuting to school and work among 6^th ^grade school children and their parents in Norway.

**Methods:**

A total of 106 pupils and 77 parents completed a questionnaire two times, 14 days apart. The questionnaire consisted of frequency items on how often the participants walk, cycle, go by car and go by public transportation to school (pupils) or work (parents). The questionnaire was divided into seasons and to/from school or work in order to cover seasonal and topographic variations. The average number of trips for each mode of commuting was calculated. Then the sample was categorised into mode of commuting: walkers, cyclists, car commuters or public transport commuters.

**Results:**

The average numbers of trips did not differ for any of the commuting modes comparing test to retest data for any of the seasons. Test-retest correlation coefficients were high for all modes of commuting (Spearman correlation coefficient were 0.85-0.92 for pupils and 0.82-0.95 for parents). Most participants categorised into mode of commuting were categorized into the same mode at both time points (97% and 95% for pupils and parents respectively).

**Conclusion:**

This newly developed questionnaire appears to be a reliable tool for measuring active commuting to school and work.

## Background

Active commuting (e.g. walking or cycling) are behaviours that might benefit both population [1] and planetary [2] health. A meta-analytic review stated recently that active commuting (among adults) was associated with an 11% reduction in cardiovascular risk [3]. An ecological study assessing the association between active commuting and obesity in Europe, North America and Australia reported that the countries with the highest prevalence of active commuting also were the countries with the lowest prevalence of obesity [4], and a study from China reported that those who acquired a car between 1989 and 1997 gained 1.8 kg in body weight compared to those not acquiring a car in the same period [5].

Cycling for transportation has been reported to be associated with lower risk of all-cause mortality [6,7]. Active commuting to worksite has also been reported to be negatively associated with being overweight [8,9]. Some studies have reported significant associations between active commuting to school and weight status among adolescents [10-13], however other studies do not find this association [14].

Studies assessing active commuting to school or worksite usually use rather simple questionnaires. Often is mode of commuting reported with a single questionnaire item only, asking for main mode of transportation to school/worksite, e.g. [8,9,11,13]. Different modes of active commuting (i.e. walking and cycling) are also usually grouped together into one category, even if walking and cycling might have different impact on health [1]. Also, in some countries there are seasonal as well as topographical variations that are seldom taken into account.

The purpose of this study is therefore to present test-retest reliability of a new questionnaire measuring frequencies of different modes of commuting to school in Norway taking seasonality and topography into account, and a similar questionnaire measuring their parent's commuting to work.

## Methods

All pupils (N = 152) in sixth grade (11-12 year olds) at four schools in the city of Kristiansand, Norway, were invited to participate in this test-retest study in the spring of 2008. The four schools were chosen from both higher and lower social status areas within Kristiansand. A questionnaire was developed to measure the frequency of different modes of commuting to school (for 6th grade pupils) and to work (for their parents) within the project Active Transportation to school and work in Norway (ATN). The questionnaire were partly based on the commuting part of the ENDORSE questionnaire [15], and inspired by the questionnaire in the Norwegian HEIA project . The questionnaires were pilot-tested on 36 pupils and ten parents. Questionnaires were administered twice to participating pupils in the classroom, each time in the presence of a trained project worker. The time period between administrations of the two questionnaires was 14 days. At both occasions the participating pupils brought home a similar questionnaire to be completed by one of the parents. Parents were informed that it was preferable that the same parent responded to the questionnaire at both times. Informed consent was sought from pupils and parents. Research clearance was obtained from the Norwegian Social Science Data Services. A total of 106 pupils (63% girls; participation rate 70%) and 81 parents (79% females) completed the questionnaire at both time points and constitute the study sample of the present study.

In a matrix [see Additional file 1], the pupils filled out how many days a week they normally (1) walk, (2) cycle, are driven by (3) car or (4) bus to and from school during fall, winter and spring. Each row had to add up to 5 days/week (days attending school). The to school, and from school, variables were added giving the number of trips walking, cycling, car- and bus commuting within each specific season (score ranging from 0 to 10). Then the mean yearly values were calculated for all seasons together (score ranging from 0 to 10), giving a total of four variables presenting the mean number of trips per week for each of the four different modes of transportation. Based on the average number of trips/week the pupils were categorised into one specific mode of commuting if more than 50% of the trips were conducted by that specific mode. If mean number of trips did not count up to 10 (trips to and from school in a normal school week) they were not categorised into mode of commuting.

A similar matrix was made for their parents commuting to work, but each row did not have to count up to five (some people normally work less or more than five days a week), and a summer season was included. Parents were also asked whether they worked away from home, and those not working away from home (n = 4) were excluded from the analyses.

All data were analysed using SPSS version 16. The data were generally skewed, hence nonparametric statistical methods were chosen. Wilcoxon signed-rank test was used to test for differences between the test and the retest estimates. Spearman correlation coefficient was used to estimate the rank order agreement between the test and the retest. Cross tabulations and Cohen's kappa coefficient was used to estimate the correct classification based on the categorisation into main mode of commuting.

## Results

The average numbers of trips did not differ for any of the commuting modes comparing test to retest data for any of the seasons or the average variables (p-values ranged from 0.08 to 1.00 for the pupils and from 0.13 to 1.00 for the parents, Table 1).

**Table 1:** Test-retest reliability of self reported frequency of different modes of commuting to school (pupils) and work (their parents).

	**Walking**			**Cycling**			**By car**			**By public transportation**		
	**test**	**retest**	**p-value**	**test**	**retest**	**p-value**	**test**	**retest**	**p-value**	**test**	**retest**	**p-value**
***Pupils***												
Fall												
median	2.0	2.0	0.79	6.0	6.0	0.89	0.0	0.0	0.08	0.0	0.0	0.80
mean	3.9	3.9		5.6	5.6		0.3	0.2		0.2	0.2	
												
Winter												
median	8.0	8.0	0.85	0.0	0.0	0.76	0.0	0.0	0.71	0.0	0.0	0.83
mean	6.8	6.7		1.4	1.7		1.1	1.0		0.6	0.5	
												
Spring												
median	0.0	0.0	0.67	10.0	8.0	0.32	0.0	0.0	0.47	0.0	0.0	1.00
mean	3.2	3.3		6.6	6.4		0.1	0.2		0.1	0.1	
												
Average												
median	4.0	3.3	0.85	5.3	5.3	0.63	0.0	0.0	0.58	0.0	0.0	0.91
mean	4.7	4.6		4.5	4.6		0.5	0.4		0.3	0.2	

***Parents***												
Fall												
median	0.0	0.0	0.28	0.0	0.0	0.73	8.0	8.0	0.17	0.0	0.0	0.29
mean	0.8	0.5		1.3	1.3		6.2	6.3		0.6	0.6	
												
Winter												
median	0.0	0.0	0.59	0.0	0.0	0.79	8.0	8.0	0.23	0.0	0.0	0.13
mean	0.9	0.7		0.5	0.4		6.5	6.6		0.9	1.1	
												
Spring												
median	0.0	0.0	0.32	0.0	0.0	0.86	7.0	8.0	0.90	0.0	0.0	1.00
mean	0.7	0.4		1.7	1.7		5.8	6.1		0.7	0.6	
												
Summer												
median	0.0	0.0	0.32	0.0	0.0	0.43	6.0	6.0	0.62	0.0	0.0	0.85
mean	0.8	0.4		1.8	20		5.5	5.7		0.7	0.6	
												
Average												
median	0.0	0.0	0.42	0.0	0.0	0.55	7.0	7.0	0.26	0.0	0.0	0.40
mean	0.8	0.5		1.3	1.4		6.0	6.2		0.7	0.7	

Figures are number of trips per week, p-values are based on Wilcoxon signed-rank test.

Test-retest correlation coefficients were high for all modes of commuting (data not presented in any table). For the pupils, Spearman correlation coefficient was 0.92 for walking, 0.92 for cycling, 0.85 for car commuting and 0.88 for public transport commuting. For the parents, Spearman correlation coefficient was 0.82 for walking, 0.86 for cycling, 0.95 for car commuting and 0.86 for public transport commuting. Correlation coefficients for the different modes within the different seasons were also all high, and all p-values were less than 0.001.

A total of 19 pupils and 14 parents were not classified into mode of commuting at one or both time points. Most participants were categorized into the same mode at both time points; kappa coefficients were 0.93 and 0.88, and percent agreement were 97% and 95%, for pupils and parents respectively (Table 2).

**Table 2:** Classification into major mode of commuting (more than 50% of trips) to school (pupils) and work (parents).

	**RETEST**				
**TEST**	**Walkers**	**Cyclist**	**Car commuters**	**Public transport commuters**	**Total**
***Pupils***					
Walkers	34	2	0	0	36
Cyclists	1	50	0	0	51
Car commuters	0	0	0	0	0
Public transport commuters	0	0	0	0	0
Total	35	52	0	0	87
					
***Parents***					
Walkers	3	1	0	0	4
Cyclists	0	6	1	0	7
Car commuters	0	1	47	0	48
Public transport commuters	0	0	0	4	4
Total	3	8	48	4	63

Test-retest Cohen's Kappa coefficients were 0.93 for pupils and 0.88 for parents.

## Discussion

The questionnaire appears to have good test-retest reliability for both assessing frequency of different commuting modes to school and to categorise the adolescents into main mode of commuting. The similar parent questionnaire also appears to be reliable for assessing commuting to work among their parents. However, a few participants were not classified due to inconsistent reports on the questionnaire, or because they did not have any main mode of commuting.

Only a few studies have reported test-retest reliability of active commuting to school [16] or worksite. In a study of 54 8-11 year olds in the USA, the kappa coefficient was 0.96 and the percentage agreement was 98.1% on two consecutive school days [17]. The question asked was *How did you get to school today? *Option were (1) bus, (2) car or truck, (3) walked, (4) bike, (5) skateboard, scooter, rollerblade, other. A second study of about 120 13-14 year olds in England reported 14 days test-retest reliability [17]. Kappa coefficients were 0.84 to 0.87 for the test-retest reliability of the children's *main part of their journey to school *(options were walking, car, bicycle, bus, train, or other). A third study including 79 12 year olds in France reported a 1 month test-retest intraclass correlation of 0.79 [18]. A single question assessed *the time used commuting to school *(none, 0-20 min/day, > 20 min/day). A fourth study on 480 girls age 10-15 in USA assessing 12 days test-retest reliability, the kappa coefficient was 0.60 and percentage agreement was 74% on the single question *how many days in the past week did you walk, bike or skate to school? *(options were none, 1 day, 2-3 days, 4 days, or every day) [19]. A fifth study from Belgium on 33 12-18 year olds reported 1 week test-retest on *time spent actively commuting to and from school *had an ICC of 0.84, a kappa coefficient of 0.53 and a percent agreement of 69% [20]. In a study among Dutch adults Wendel-Vos and colleagues [21] reported Spearman r's of 0.72 and 0.96, respectively for the time spent walking and cycling to worksite. In general, these studies report good test-retest reliability of self-reported measures on commuting to school and worksite. However, most studies only use single questionnaire items assessing main mode of commuting, or they report a combined measure of time spent on combined active commuting (i.e. walking and cycling together).

The strength of the present study is the comprehensive design of the measure on commuting to school and work, making it possible to assess the frequency of the different modes of active commuting to and from school and work in the different seasons. The results show that such a comprehensive questionnaire is statistical reliable among 6^th ^graders and their parents.

A limitation of the present study is that the schools are probably not representative for the general Norwegian population. In the present study, none pupils were categorised as car or buss commuters. In a national survey it was reported that 43% did go to school by car or bus on the survey day [22]. This deviation might be explained by the schools included in the study being situated in suburbs with local schools surrounding Kristiansand city. Also, more girls than boys and mothers than fathers participated in the study, and the participation among the parents were 76% of participating pupils and 54% of eligible pupils, raising question about generalizability, especially among the parents. However, the objective of this study was to present a new methodology and to assess the reliability of the questionnaire. The reliability results presented are not supposed to be influenced by the study design and participation.

## Conclusion

This study has presented the newly developed ATN questionnaire, which makes it possible to assess the frequency of the different modes of active commuting to and from school and work in the different seasons. The questionnaire appears to be a reliable tool for measuring active commuting to school and work in Norway, and modified versions might be used by research and practice in other places.

## Competing interests

The authors declare that they have no competing interests.

## Authors' contributions

EB and LAB conceived and designed the study together. EB analysed the data and drafted the manuscript. LAB collected and processed the data, and revised the manuscript critically. Both authors have read and approved the final manuscript.

## Supplementary Material

Additional file 1 **Questionnaire matrix for reporting frequencies of different modes of commuting to school**. The matrix is an English translation of the ATN questionnaire measuring frequencies of different modes of commuting to school. Click here for file
