# Supplementary material for: Test-retest reliability of a new self reported comprehensive questionnaire measuring frequencies of different modes of adolescents commuting to school and their parents commuting to work - the ATN questionnaire
Source: Int J Behav Nutr Phys Act. 2009 Oct 12;6:68. doi: 10.1186/1479-5868-6-68 (PMC2768672; doi:10.1186/1479-5868-6-68)
Supplement: Additional file 1 — Questionnaire matrix for reporting frequencies of different modes of commuting to school. The matrix is an English translation of the ATN questionnaire measuring frequencies of different modes of commuting to school. [file 1479-5868-6-68-S1.PDF]

How do you usually get to and from school? Write down the number of days for every season. For every line the total must add up to 5.

**Season**		**Walk**	**Cycle**	**By car**	**By public transportation**	**Total**
**Fall** (Sept-Nov)	To school					=5 days
	From school					=5 days
**Winter** (Dec-Feb)	To school					=5 days
	From school					=5 days
**Spring** (Mar-May)	To school					=5 days
	From school					=5 days
